# Clinical Diagnosis of Gastric Cancer by High-Sensitivity THz Fiber-Based Fast-Scanning Near-Field Imaging

**DOI:** 10.3390/cancers14163932

**Published:** 2022-08-15

**Authors:** Hua Chen, Juan Han, Shihua Ma, Xiao Li, Tianzhu Qiu, Xiaofeng Chen

**Affiliations:** 1 School of Physics, Southeast University, Nanjing 211189, China; 2 Department of Oncology, The First Affiliated Hospital of Nanjing Medical University, Nanjing 210029, China; 3 Department of Oncology, Pukou Branch Hospital of Jiangsu Province Hospital, Nanjing 211800, China

**Keywords:** gastric cancer, absorption contrast, THz, imaging diagnosis

## Abstract

**Simple Summary:**

In order to realize rapid and complete pre-screening in the pathological examination of gastric cancer and assist in quickly determining the boundary between cancerous and healthy cells, we developed a fast-scanning near-field terahertz (THz) imaging system. This imaging system is compact, low-cost, and easy to operate. THz examination does not need any processing after sectioning, and the diagnostic results are directly displayed in images within one minute. Compared with the H&E staining method, THz imaging diagnosis uses a quantitative absorption coefficient to distinguish cancer tissue and healthy tissue, which makes the automation of the tissue sampling pre-screening procedure possible and will save valuable time to help quickly define cancer tissue. At the same time, the spatial resolution of our near-field imaging system reaches λ/17. Using THz imaging to accurately define the margins of cancer can not only conserve healthy tissues but also minimize the number of second surgical procedures, which would save a lot of additional hospital resources.

**Abstract:**

The distinguishable absorption contrast among healthy gastric tissues, carcinoma in situ and cancer tissues in the THz frequency range is one of the keys to realizing gastric cancer diagnosis by THz imaging. Based on microwave devices and a sub-wavelength fiber, we developed a fast-scanning THz imaging system combined with the principle of surface plasmon resonance enhancement. This imaging system has a near-field λ/17 spatial resolution and imaging S/N ratio as high as 10^8^:1, and the image results are directly displayed within 1 min. We also successfully demonstrated the image diagnostic capability on sliced tissues from eight patients with gastric cancer. The results indicate that THz absorption images can not only clearly distinguish cancer tissue from healthy tissues but also accurately define the margins of cancer. Through a medical statistical study of 40 sliced tissues from 40 patients, we prove that THz imaging can be used as a standalone method to diagnose gastric cancer tissues with a diagnostic specificity and sensitivity of 100%. Compared with the H&E staining method, THz imaging diagnosis makes the automation of tissue-sampling pre-screening procedure possible and assists in quickly determining the boundary between cancerous and healthy tissues.

## 1. Introduction

Gastric cancer is a malignant tumor originating from the gastric mucosal epithelium and is one of the major diseases endangering people’s health. In 2020, more than 1 million new cases of gastric cancer were diagnosed worldwide, accounting for 5.6% of all new cancer cases. Meanwhile, gastric cancer causes 769,000 deaths, which accounts for 7.7% of all cancer deaths [1]. The stomach is mainly composed of muscle tissue. When healthy stomach tissue turns into cancer tissue, the metabolism accelerates. The molecular structure and important material components in the tissue, such as protein, nucleic acid, and phospholipids, will change significantly. In the process of pathological changes, the structure of the gastric tissue evolves, and the intramolecular and intermolecular water content increases. The research results in recent years have found that these changes in gastric tissues can be detected in the THz band [2,3,4,5,6,7].

THz radiation waves are electromagnetic radiation between infrared light and microwave, with a frequency range between 100 GHz and 100 THz and a corresponding wavelength between 3 μm and 3 mm. The THz wavelength is longer than visible light, resulting in lower scattering in biological tissues and deeper penetration depth of samples [8,9]. THz waves are nonionizing radiation. Their photon energy is smaller than that of general X-ray and visible light, and they do not harm biological samples [10,11]. At the same time, the natural rotational or vibrational energy levels of many molecules fall in the THz wave band, and the molecules will resonantly absorb the THz wave [12,13,14,15,16]. Therefore, THz waves can be directly used to study molecular distribution and to provide soft-tissue-detection sensitivity higher than that of X-rays [17,18].

In our previous works, we have proved that THz images can correctly diagnose human cancer tissues [7,19,20,21,22]. Herein, to realize the rapid and complete pre-screening of gastric cancer and assist in quickly determining the boundary between cancerous and healthy tissues, we improved on our original near-field THz imaging system by using a bull’s-eye metallic spatial filter with a subwavelength bow-tie-shaped aperture (referred to as a bow-tie filter) and a highly sensitive cryogenic-temperature-operated Schottky diode detector. The spatial resolution in the near-field region increased from λ/4 to λ/17, and the signal-to-noise (S/N) ratio of the imaging system increased 1000 times. We then used this near-field imaging system to diagnose unstained gastric tissue slices without dehydration. We chose 108 GHz as the working frequency. The absorption coefficient contrast at 108 GHz was first confirmed by 60 gastric tissues from 40 patients, including 20 cancer tissues, 20 healthy tissues, and 20 carcinoma in situ tissues. Then, according to the absorption coefficient contrast, we performed THz near-field imaging diagnosis of eight unknown tissues from eight patients, and in terms of the size and shape of the cancer tissue, the imaging results are surprisingly consistent with the subsequent identification of the same sliced sample diagnosed by pathological H&E staining.

## 2. Materials and Methods

### 2.1. Sample Preparation

All of the investigated tissues were surgically removed, stored, hydrated, and frozen in the Department of Pathology, First Affiliated Hospital, Nanjing Medical University, Nanjing, Jiangsu, China. In a THz spectra acquisition study, we collected 60 gastric tissues from 40 patients, including 20 cancer tissues, 20 healthy tissues, and 20 carcinoma in situ tissues. Samples were prepared with a uniform 500 μm thickness by cutting slices of fresh tissue. In a THz image diagnosis study, we collected 8 gastric tissues from 8 patients. The surgically removed tissues under investigation were stored frozen (without dehydration) immediately after the surgical procedure at −30 °C. Before THz imaging examination, we frozen-sectioned the tissues into 20 μm-thick slices and placed them on the selected 150 μm-thick microscope coverglasses. In a medical statistical study, we collected 40 gastric tissues from 40 patients.

### 2.2. Experimental Setup

**Figure 1:**
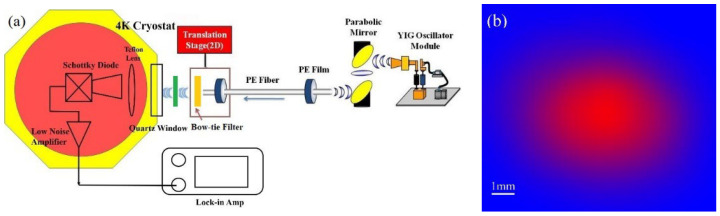
(**a**) Schematic diagram of the THz near-field imaging system. (**b**) A direct 2D 10 mm × 10 mm scanning image of air.

Our previous THz spectroscopy study of gastric tissues revealed that THz absorption decreases at lower frequencies, and the difference in absorption between healthy gastric tissues, carcinoma in situ, and cancer tissues is clear [7], so we used a frequency of 108 GHz for imaging measurements. A schematic diagram of the THz near-field imaging system is shown in Figure 1a. The working principle of the imaging system remains the same as that described in our previous system [19,20]. Briefly, a YIG oscillator module is used to radiate a 108 GHz THz wave, and then we collect the THz wave with a pair of off-axis parabolic mirrors and then focus it into the PE sub-wavelength fiber. The parameter of the fiber is 600 μm in diameter and 40 cm in length. Behind the fiber output, we used a bow-tie filter to achieve high enhanced transmission power and near-field spatial resolution [23,24,25]. We also used a cryogenic-temperature-operated Schottky diode detector to improve the detection sensitivity. The working temperature of the detector is approximately 4 K. [22]. Finally, the collected signals were analyzed with a lock-in amplifier. The image shown in Figure 1b is obtained by two-dimensional (2D) 10 mm × 10 mm direct scanning of the output end of the fiber with an imaging time less than 1 min (100 × 100 pixels). The results show the S/N ratio of the imaging system to be approximately 10^8^:1, which is an improvement of approximately 10^3^ times compared to our previous imaging system [19].

**Figure 2:**
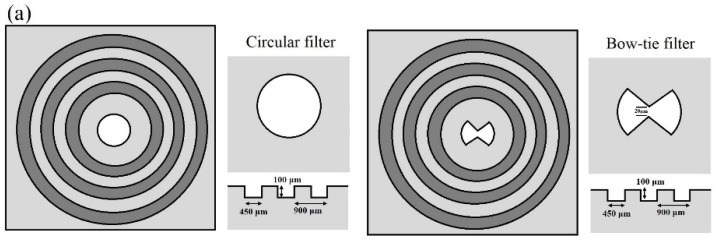

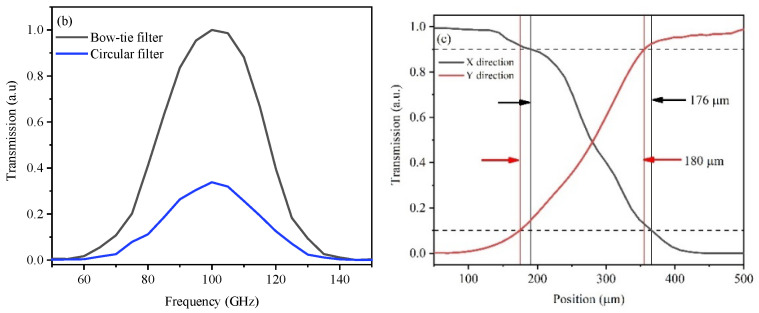
(**a**) Schematic illustration of a circular filter and a bow-tie filter. (**b**) The measured transmission-enhancement factor of the bow-tie filter normalized to the circular filter. (**c**) The measured spatial-resolution curves in the *x* and *y* directions of the bow-tie filter.

Figure 2a is a schematic illustration of a conventional circular filter and a bow-tie filter, and the diameters of the apertures are both 200 μm, about 1/5 of the incident wavelength. The surface of the periodic concentric grooves on both filters is coated with a 0.2 μm Au film, and the Au film will cause the resonant coupling of the THz waves to the surface plasmon waves. Through this design, we will obtain high incident energy and ultra-high spatial resolution after being modulated by the filtered wave plate. Figure 2b shows the measured transmission-enhancement factor of the bow-tie filter normalized to the circular filter. The transmission ratio was more than 3-fold higher. Figure 2c shows the measured spatial resolution curves for the bow-tie filter by a knife-edge method. By scanning in the X- and Y-directions of the THz energy intensity radiated through the outlet side of the bow-tie filter, the effective spatial resolution was thus quantified as 176 μm in the x-direction and 180 μm in the y-direction. The spatial resolution in the near-field region thus increased to λ/17.

## 3. Results

### 3.1. THz Absorption Spectra of Gastric Tissues

**Figure 3:**
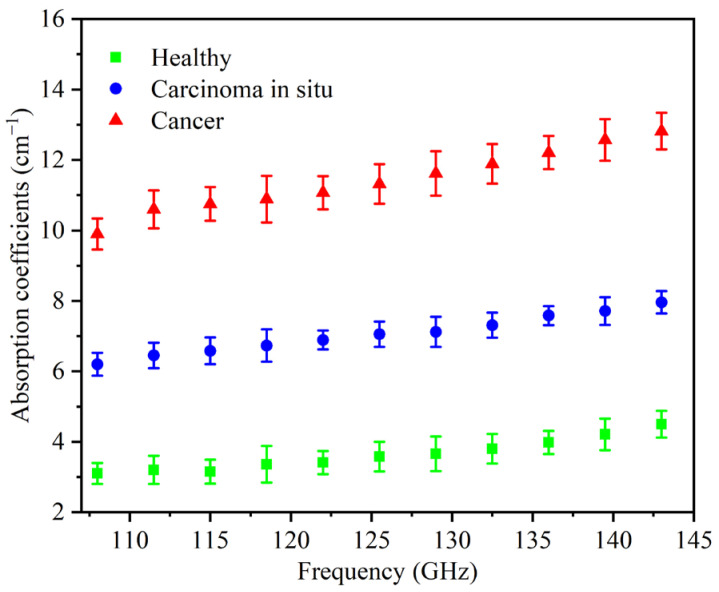
The average absorption coefficient spectra of healthy tissues (green), carcinoma in situ tissues (blue), and cancer tissues (red).

Between 108 GHz and 143 GHz, we detected the absorption spectra of 60 gastric tissues from 40 patients, including 20 healthy tissues, 20 cancer tissues, and 20 carcinoma in situ tissues and calculated the corresponding absorption coefficients (α). Figure 3 shows the range of the α of these 60 samples. The mean absorption coefficients of healthy tissues, carcinoma in situ tissues, and cancer tissues are 2.78–3.42 cm^−1^, 5.68–6.52 cm^−1^, and 9.46–10.34 cm^−1^ at 108 GHz, respectively. The values indicate that the absorption spectra can clearly distinguish these three gastric tissues, and this distinct feature is indued by the combination of water with functional groups such as protein and the increase in protein density in tissues [26,27]. The distinguishable absorption contrast is one of the keys to realizing THz diagnosis in the developed imaging system.

### 3.2. Image Diagnosis

We gained the THz images by normalizing the background to correct the angle-dependent bending loss induced by the fiber scanning, while the background was measured by scanning one blank selected coverglass. All diagnostic images of the gastric tissue sections in this work are displayed to α, which was estimated according to the Beer–Lambert law. Therefore, we calculate the α of gastric tissues as: α = ln(I_S_/I_B_)/h [28,29], where I_S_ is the transmitted power of the THz wave through the coverglass and the gastric tissue sections, while I_B_ is the background. h is the thickness of the sections. In addition, the range of α we used for analyzing the imaging results is shown in Figure 3. Therefore, in the following THz diagnostic images, we define the color bar as follows: green was marked as healthy tissue (α = 2.78–3.42 cm^−1^), while blue was carcinoma in situ (α = 5.68–6.52 cm^−1^), and red was cancer regions (α = 9.46–10.34 cm^−1^).

**Figure 4:**
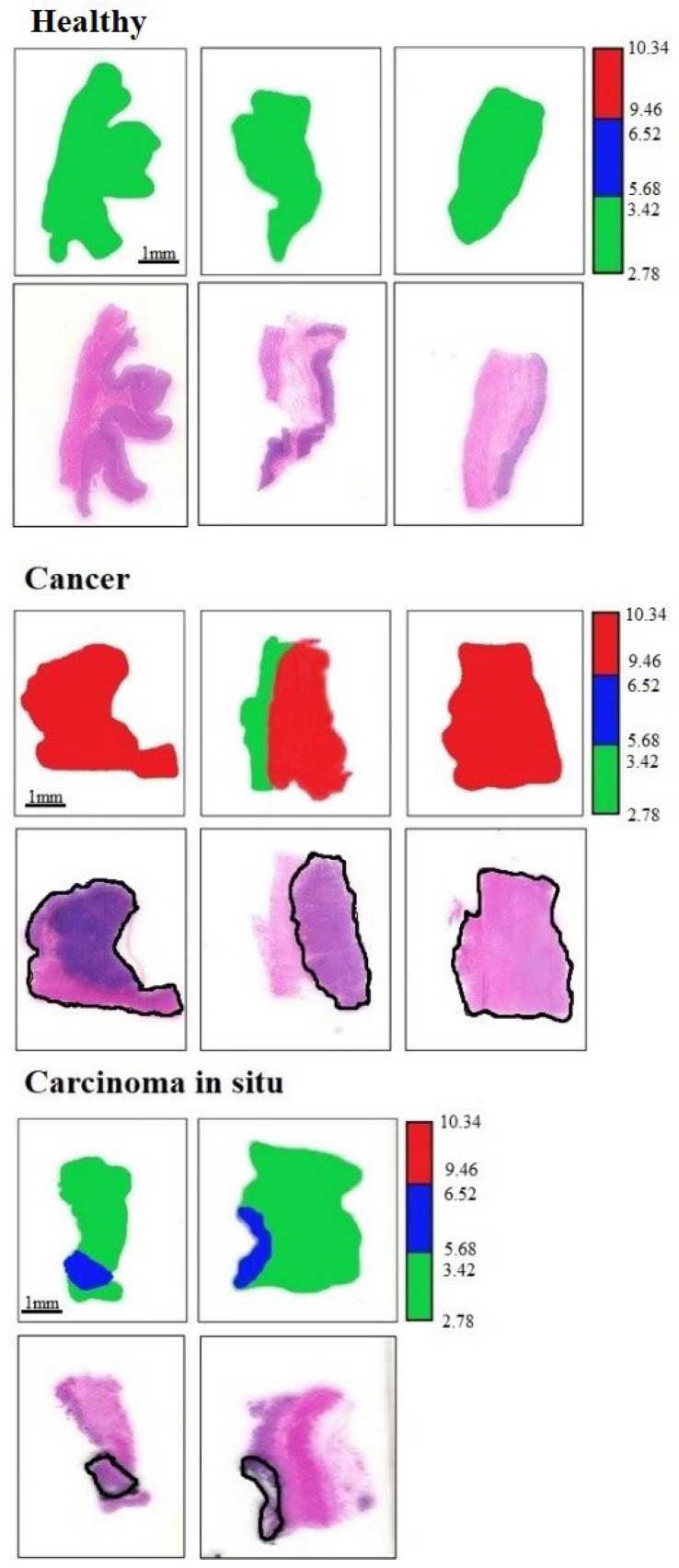
THz near-field images of 3 healthy tissues, 2 carcinoma in situ tissues, and 3 cancer tissues and the corresponding pathologic photomicrograph of H&E-stained sections.

At first, we investigated the capability of THz imaging to diagnose gastric cancer tissues, and eight unknown gastric tissues were selected. The whole process of THz imaging diagnosis is carried out independently. That is to say, we did not know the properties of gastric tissues before THz imaging. After imaging, we sent all specimens for clinical pathological H&E staining and examination in the Department of Pathology, the First Affiliated Hospital. During THz imaging, the temperature and humidity of the laboratory were maintained at 23 °C and ~50%. The acquired THz diagnosis images and the corresponding pathologic photomicrograph of H&E-stained sections are shown in Figure 4. According to the corresponding THz diagnosis images, we successfully identified these eight unknown samples as three healthy tissues, two carcinoma in situ, and three cancer tissues. By comparing this with pathological staining photos, we not only proved the accuracy of THz imaging in the diagnosis of gastric cancer but also showed that the size and shape of the gastric tissues were consistent with pathological results. We also marked the areas of carcinoma in situ and cancer tissues in the pathological staining photos with a black solid boundary. The shape and the location of the cancer regions were found to excellently agree with THz diagnosis images.

### 3.3. Medical Statistical Research

With a high THz diagnostic capability, we further studied its diagnostic performance through medical statistical methods. In clinical diagnosis, negative or positive corresponds to health or disease, respectively. However, diagnostic results may be misdiagnosed. Therefore, we can divide the diagnostic results as true negative (TN), false negative (FN), true positive (TP), and false positive (FP). Finally, the corresponding diagnostic specificity and sensitivity can be expressed as: *Specificity* = TN/(TN + FP) and *Sensitivity* = TP/(TN + FN) [30,31]. In the following statistical study, we used two sample slices per patient on the additional 40 unknown samples from 40 patients. One was used for the THz imaging diagnosis, and the other was used for clinical H&E staining. The corresponding diagnostic results are shown in Table 1. The results of these two diagnostic methods agree clearly, and the corresponding calculation results of specificity and sensitivity are both 100%. The results also indicate that THz imaging can be used as a standalone method to diagnose gastric cancer tissues.

## 4. Discussion

For the future application of THz imaging methods to diagnose gastric cancer in clinical practice, we demonstrated their diagnostic capability with three types of gastric tissues. The diagnosis of gastritis and gastric polyp tissue samples is also in our research plan, but due to the lack of samples, we cannot achieve this. However, the structure and water content of different tissue samples will lead to different degrees of THz absorption, and we believe that absorption contrast can be used to distinguish gastric tissues with different degrees of pathological changes. At the same time, using absorption contrast to identify different tissues is very sensitive to the thickness change of tissue slices. According to the Beer–Lambert law, we found that if the sample thickness fluctuation exceeded 5 μm, the calculated α would be fallacious. Man-made or machine causes would both induce this poor section quality condition. In this paper, we report our results with a thickness fluctuation less than 2 μm. Based on the same considerations, before sectioning the tissues, we selected the coverglasses carefully with the same thickness (150 μm), and then all of the selected coverglasses were sent for THz transmission measurement. After acquiring the transmission power of the 150 μm-thick coverglasses, we removed the coverglasses whose transmission power was 4% lower or higher than the average value. It is important to notice that 4% transmission fluctuation will mean 2.0 mm^−1^ error in the measured α for a 20 μm-thick sample slice.

In the current hospital clinical diagnosis, the methods of diagnosing gastric cancer mainly include endoscopic biopsy, endoscopic ultrasonography, gastrointestinal X-ray barium meal examination, CT examination, etc. With the help of these diagnostic methods, doctors can directly observe the pathological changes in gastric tissue, and then determine the location and scope of the lesions. In the subsequent histopathological diagnosis, the H&E staining method is the most basic and widely used technology, which can clearly show various tissue types or cell components, as well as the general morphological and structural characteristics of lesions [7]. At present, after tissue sampling and slicing, H&E staining diagnosis still takes about 3 days, including dozens of processes such as dehydration, baking, staining, sealing, film reading diagnosis, and so on. The whole process not only needs to consume a lot of various chemical reagents but also requires high professional skills of doctors. However, THz examination does not need any processing after sectioning, and the diagnostic results are directly displayed in images within one minute. After THz examination, the sample is still intact and can be subjected to subsequent pathological analysis. Compared with the H&E staining method, THz imaging diagnosis uses the quantitative absorption coefficient to distinguish cancer tissue and healthy tissue, which makes the automation of the tissue sampling pre-screening procedure possible and will save valuable time to help quickly define cancer tissue. At the same time, the spatial resolution of our near-field imaging system reaches λ/17. Using THz imaging to accurately define the margins of cancer can not only conserve healthy tissues but also minimize the number of second surgical procedures, which would save a lot of additional hospital resources.

## 5. Conclusions

Based on microwave devices and a sub-wavelength fiber, we developed a fast-scanning THz imaging system. With two significant developments, including introducing a bow-tie filter and a cryogenic-temperature-operated Schottky diode detector, we realized a near-field λ/17 spatial resolution and imaging S/N ratio as high as 10^8^:1. The THz absorption spectra study indicated that THz absorption contrast of healthy gastric tissues, carcinoma in situ, and cancer tissues can be clearly distinguishable at 108 GHz. According to the results of the absorption contrast, we successfully proved that THz imaging can diagnose gastric cancer as a standalone method. Compared with the H&E staining method, the quantitative THz absorption coefficient is used to distinguish the type of tissues, and the diagnostic results are directly displayed in images, which would make the automation of a tissue diagnosis procedure possible.

## Figures and Tables

**Table 1:** Diagnostic results via the THz imaging and H&E staining methods.

No.	THz Imaging	H&E	Results
**1**	Healthy	Healthy	TN
**2**	Cancer	Cancer	TP
**3**	Cancer	Cancer	TP
**4**	Cancer	Cancer	TP
**5**	Healthy	Healthy	TN
**6**	Healthy	Healthy	TN
**8**	Carcinoma in situ	Carcinoma in situ	TP
**9**	Carcinoma in situ	Carcinoma in situ	TP
**10**	Carcinoma in situ	Carcinoma in situ	TP
**11**	Cancer	Cancer	TP
**12**	Healthy	Healthy	TN
**13**	Healthy	Healthy	TN
**14**	Cancer	Cancer	TP
**15**	Healthy	Healthy	TN
**16**	Carcinoma in situ	Carcinoma in situ	TP
**17**	Carcinoma in situ	Carcinoma in situ	TP
**18**	Healthy	Healthy	TN
**19**	Healthy	Healthy	TN
**20**	Healthy	Healthy	TN
**21**	Carcinoma in situ	Carcinoma in situ	TP
**22**	Cancer	Cancer	TP
**23**	Cancer	Cancer	TP
**24**	Healthy	Healthy	TN
**25**	Healthy	Healthy	TN
**26**	Carcinoma in situ	Carcinoma in situ	TP
**27**	Cancer	Cancer	TP
**28**	Cancer	Cancer	TP
**29**	Cancer	Cancer	TP
**30**	Cancer	Cancer	TP
**32**	Carcinoma in situ	Carcinoma in situ	TP
**33**	Healthy	Healthy	TN
**34**	Healthy	Healthy	TN
**35**	Carcinoma in situ	Carcinoma in situ	TP
**36**	Carcinoma in situ	Carcinoma in situ	TP
**37**	Carcinoma in situ	Carcinoma in situ	TP
**38**	Cancer	Cancer	TP
**39**	Healthy	Healthy	TN
**40**	Healthy	Healthy	TN

## Data Availability

The data that support the findings of this study are available on request from the corresponding author.
